# An analysis of pharmacists' workplace patient safety perceptions across practice setting and role characteristics

**DOI:** 10.1016/j.rcsop.2021.100042

**Published:** 2021-06-29

**Authors:** Reginald Dilliard, Nicholas E. Hagemeier, Brady Ratliff, Rebecca Maloney

**Affiliations:** a Tennessee Board of Pharmacy, 665 Mainstream Dr, Nashville, TN 37243, United States of America; b East Tennessee State University Gatton College of Pharmacy, Johnson City, TN, United States of America

**Keywords:** Community pharmacy, Workplace safety, Patient safety, Community pharmacist

## Abstract

**Background:**

Lay press investigations have been published that describe pharmacist errors and the workplace environment in the community pharmacy setting. However, recent studies that explore pharmacists' perceptions of patient safety in the workplace are limited.

**Objectives:**

1) To describe pharmacists' perceptions of workplace patient safety; 2) To compare pharmacists' perceptions of workplace patient safety across practice setting type, pharmacist roles, average hours worked per shift, and average hours worked per week.

**Methods:**

Actively licensed Tennessee pharmacists were recruited from January 1 and June 30, 2019 to complete a 13-item survey of workplace patient safety perceptions (***N***=1391). Descriptive statistics were calculated, and nonparametric statistical tests employed to compare differences in perceptions across practice setting type, pharmacist roles, and hours worked per shift and per week.

**Results:**

Statistically significant differences in workplace patient safety perceptions were noted across practice setting type (p values <.001) and pharmacist roles (p values <.001). The extent to which pharmacists agreed/strongly agreed that their employer provides a work environment that allows for safe patient care ranged from 29.7% of chain community pharmacists to 85% of compounding pharmacists. Fifty-two percent of staff pharmacists, 56.5% of relief pharmacists, and 58.5% of managers/pharmacists in charge agreed or strongly agreed that their employer provides a work environment that allows for safe patient care, whereas 89.3% of regional managers/directors/vice-presidents and 72.5% of clinical/specialty pharmacists indicated the same. Average hours per shift was inversely correlated with perceptions of workplace patient safety (p values <.001).

**Conclusion:**

Tennessee pharmacists' perceptions of workplace patient safety varied widely across practice setting type and pharmacist roles. Perceptions of safety were notably lower in the chain community pharmacy setting. Additional research is warranted to better understand the relationship between pharmacist perceptions and quantifiable patient safety metrics, particularly in the chain community pharmacy setting.

## 1 Introduction

Licensed pharmacists are intimately involved in the healthcare system across multiple settings, including but not limited to hospitals, primary care clinics, long-term care facilities, and community pharmacies. As medication experts, pharmacists collaborate interprofessionally to optimize pharmacotherapeutic plans, promote patient safety through prospective drug utilization review (DUR) processes, and routinely serve as the final verification step in dispensing prescription drugs to patients. Recent evidence indicates health professionals are increasingly experiencing job stress, moral distress, high workload, and other potentially negative realities and/or perceptions that contribute to decreased health professional wellbeing (e.g., increased burnout, substance use disorders, anxiety, depression).^1^, ^2^, ^3^, ^4^, ^5^, ^6^, ^7^, ^8^, ^9^, ^10^ Importantly, evidence also suggests workplace factors contribute to increased medical errors and have downstream, preventable consequences on patient morbidity and mortality.^11^, ^12^, ^13^

Practicing error-free is arguably the societal and professional expectation for pharmacists.^14^ However, medication-related errors account for approximately 25% of preventable patient harm incidents.^15^ In the community pharmacy setting, peer-reviewed literature and lay press investigations alike have demonstrated that dispensing errors are relatively common.^16^, ^17^, ^18^ Flynn et al.^16^ found that over 20% of prescriptions dispensed in a secret shopper study contained dispensing errors, 3% of which had the potential to cause patient harm. A 2016 *Chicago Tribune* investigation noted that community pharmacists dispensed potentially harmful drug combinations to patients in approximately half of secret shopper attempts to get the medications.^17^ A recent *New York Times* article brought renewed attention to errors occurring in community pharmacy settings and the perceptions of community pharmacists.^18^ Succinctly, workplace demands, either real or perceived, are routinely contributing factors to dispensing errors and quality of patient care.^13^^,^^14^^,^^17^, ^18^, ^19^, ^20^, ^21^, ^22^

While the causal nature of workplace conditions and pharmacist errors and subsequent patient harm is difficult to substantiate and quantify, the emotion-laden response to patient harm deservedly invites policy reform to protect public health. As a self-regulating profession with a majority of influence at the state level, boards of pharmacy are simultaneously called to protect patients from harm and pharmacists from workplace conditions that compromise patient safety. We conducted a brief survey of licensed pharmacists in the State of Tennessee to determine their perceptions of the current workplace environment with explicit emphasis on patient safety. Given verbal communication received from pharmacists employed in the community pharmacy setting, coupled with recent lay press investigations, we were particularly interested in understanding differences in workplace perceptions across pharmacy practice setting type and pharmacist roles.

## 2 Methods

### 2.1 Participant recruitment

This anonymous, cross-sectional, survey-based study was conducted by the Tennessee Board of Pharmacy from January to June 2019. Actively licensed Tennessee pharmacists were recruited to participate in the study through multiple mechanisms. First, paper-based surveys were given to pharmacists by ten Board of Pharmacy investigators during routine practice site inspections. It is estimated that 700-800 practice site visits were conducted during the study period. Surveys were also disseminated to attendees at eight continuing pharmaceutical education events conducted throughout the state, at which the Executive Director of the Board of Pharmacy provided a legislative update. Finally, surveys were disseminated to pharmacists who attended the Tennessee Pharmacists Association Winter Meeting in February 2019, at which the Executive Director of the Board also provided a legislative update. This multi-pronged recruitment approach facilitated in-person delivery and same-day collection of the survey instruments from respondents. However, pharmacists also could complete the questionnaire at their leisure and mail/fax the completed questionnaire to the Board office. Additionally, dissemination at conferences and meetings allowed for completing the survey instrument outside the workplace setting and in an anonymous manner. Finally, dissemination via practice site inspections allowed for consistent, in-person delivery of the survey instrument to pharmacists across all practice sites inspected. Participation was voluntary, and no identifiable information was collected from pharmacists. Surveys completed by pharmacy technicians, non-pharmacists, and pharmacists not actively engaged in practice (e.g., inactive license, retired) were excluded from the analysis.

A total of 1,391 usable surveys were collected from actively licensed, practicing Tennessee pharmacists. Given the recruitment methodology used, it was not possible to determine the overall response rate for the study. For context, approximately 13,200 pharmacists are actively licensed with the Tennessee Board of Pharmacy, a number that includes pharmacists not currently practicing and those practicing in other states. At a minimum, the responses obtained in this study represent 10.5% of pharmacists actively practicing in Tennessee.

### 2.2 Data collection and analysis

The Tennessee Board of Pharmacy developed a 13-item survey instrument at the request of Board of Pharmacy investigators. The Board adapted five workplace safety items (I feel I have adequate time to complete my job in a safe and effective manner; I feel that my employer has provided a work environment that allows for safe patient care; I feel there is adequate technician staffing at my practice site to provide a safe environment for patient care; I feel there is adequate pharmacist staffing at my practice site to provide a safe environment for patient care; I am given the opportunity to take lunch breaks or time away from the pharmacy in my practice) from a survey instrument developed and used by the Oregon Board of Pharmacy to assess pharmacists' perceptions of patient safety in the workplace.^23^ Three additional items (I feel the workload to staff ratio allows me to provide for patients in a safe manner; I feel pressured or intimidated to meet standards or metrics that may interfere with safe patient care at my practice site; I am happy with my current practice site and working environment) were developed based on verbal communication from Tennessee pharmacists and technicians regarding patient safety in the workplace. All workplace perception items were responded to using a 5-point Likert response scale (1=strongly disagree; 5=strongly agree), the same response scale used in the Oregon study, and a subsequent study that employed adaptations of the Oregon study items.^21^^,^^23^ As mentioned in the limitations section, no psychometric analyses have been conducted on the workplace perception items. Four items were developed to elicit pharmacists' workplace characteristics, including pharmacist role, practice setting type (e.g., community-chain, community-independent, inpatient hospital), average hours worked per shift, and average hours worked per week. One concluding open-ended item was included in the survey instrument to elicit pharmacists' perceptions but was not analyzed for this manuscript.

Once developed by the Board, the survey instrument was pre-tested with Board investigators to determine item clarity, relevance, and comprehensiveness of the verbal communication they were receiving from pharmacists and pharmacy technicians. With Board investigator approval, the survey instrument was disseminated as previously described. The survey instrument is included in the Appendix.

Survey data were entered into a Microsoft Excel spreadsheet by the study team. SPSS v25 (IBM Corp, Armonk, NY) was used to analyze the data. In rare cases where pharmacist respondents circled more than one response (e.g., strongly agree and agree), the response closest to the midpoint of the response scale was recorded. Given the ordinal nature of the individual items that elicited perceptions of the work environment, the Kruskal Wallis test and post hoc Mann Whitney tests were used to compare differences in perceptions across practice setting type and pharmacist roles. Spearman's rho was used to examine correlations between hours worked per shift and per week and workplace perceptions. A p < .05 was considered significant for all statistical analyses. This study was considered exempt by the East Tennessee State University Institutional Review Board.

## 3 Results

**Fig. 1:**
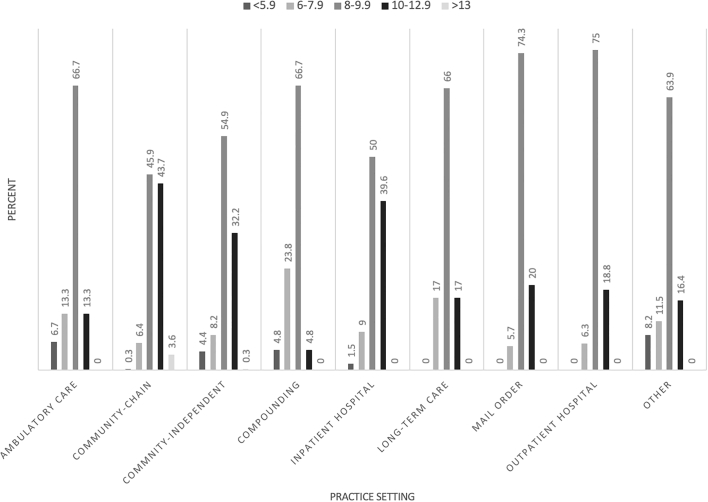
Pharmacists' reported hours per shift by primary practice setting (N=1391).

Respondent work characteristics are presented in Table 1. Overall, respondents tended to be staff pharmacists or pharmacists in charge in the chain or independent community practice setting who work, on average, 8 to 12.9-hour shifts and 30 to 49.9 hours per week. Fig. 1 presents hours per shift data across practice setting types.

**Table 1:** Respondent workplace characteristics (N=1391).

Variable		No. (%)
Practice role	Staff Pharmacist	669 (48.1)
	Pharmacist in Charge/Manager	443 (31.8)
	Clinical/Specialty Pharmacist	136 (9.8)
	Relief Pharmacist	115 (8.3)
	Regional Manager/Director/VP	28 (2.0)
Practice setting	Community – Chain	592 (42.6)
	Community – Independent	317 (22.8)
	Inpatient Hospital	269 (19.4)
	Other	61 (4.4)
	Long-Term Care	47 (3.4)
	Mail Order	35 (2.5)
	Outpatient Hospital	32 (2.3)
	Compounding	21 (1.5)
	Ambulatory Care	15 (1.1)
Hours per shift	<5.9	27 (1.9)
	6–7.9	114 (8.2)
	8–9.9	723 (52.1)
	10–12.9	501 (36.1)
	≥13	22 (1.6)
Hours per week	<30	152 (10.9)
	30–39.9	367 (26.4)
	40–49.9	718 (51.7)
	50–59.9	114 (8.2)
	≥60	38 (2.7)

Source/Notes: Authors' analysis of Tennessee Board of Pharmacy Survey, 2019.

Workplace perceptions across practice setting type are presented in Table 2. Statistically significant differences in responses were noted across practice settings (p < .001). Generally, pharmacists practicing in compounding and independent community pharmacy settings reported the most favorable perceptions of patient safety. Post hoc tests indicated respondents who practice in the chain community setting reported less positive perceptions as compared to all other practice settings for all items (p < .05). Whereas approximately 60% of chain community pharmacists disagreed or strongly disagreed that they have adequate time to complete their job in a safe and effective manner, at least two-thirds of pharmacists in every other practice setting agreed or strongly agreed with the statement. Approximately 4% of respondents in non-chain community pharmacies strongly disagreed that their employer provides a work environment that allows for safe patient care. Five times that percentage of chain community pharmacists indicated the same.

**Table 2:** Pharmacists' workplace perceptions by practice setting (N=1391)^a^.

Item	Response^b^	Practice setting^c^								
		Amb. Care (*N* = 15)	Comm – Chain (*N* = 592)	Comm – Indep. (*N* = 317)	Compound. (*N* = 21)	Inpatient Hospital (*N* = 269)	LTC (*N* = 47)	Mail Order (*N* = 35)	Outpatient Hospital (*N* = 32)	Other (*N* = 61)
I feel I have adequate time to complete my job in a safe and effective manner	SA	4 (26.7)	23 (3.9)	82 (25.9)	8 (38.1)	50 (18.6)	7 (14.9)	4 (11.4)	4 (12.5)	11 (18.0)
	A	8 (53.3)	138 (23.3)	147 (46.4)	10 (47.6)	128 (47.6)	24 (51.1)	20 (57.1)	20 (62.5)	33 (54.1)
	N	1 (6.7)	83 (14.0)	37 (11.7)	1 (4.8)	37 (13.8)	9 (19.1)	5 (14.3)	4 (12.5)	7 (11.5)
	D	1 (6.7)	215 (36.3)	45 (14.2)	2 (9.5)	42 (15.6)	4 (8.5)	4 (11.4)	2 (6.3)	7 (11.5)
	SD	1 (6.7)	133 (22.5)	6 (1.9)	0 (0)	12 (4.5)	3 (6.4)	2 (5.7)	2 (6.3)	3 (4.9)
I feel that my employer has provided a work environment that allows for safe patient care	SA	6 (40.0)	38 (6.4)	136 (43.0)	9 (45.0)	71 (26.4)	11 (23.4)	4 (11.4)	7 (21.9)	19 (31.1)
	A	6 (40.0)	138 (23.3)	143 (45.3)	8 (40.0)	116 (43.1)	22 (46.8)	20 (57.1)	17 (53.1)	25 (41.0)
	N	0 (0)	119 (20.1)	19 (6.0)	1 (5.0)	31 (11.5)	10 (21.3)	7 (20.0)	5 (15.6)	11 (18.0)
	D	3 (20.0)	186 (31.4)	18 (5.7)	2 (10.0)	41 (15.2)	3 (6.4)	3 (8.6)	3 (9.4)	5 (8.2)
	SD	0 (0)	111 (18.8)	0 (0)	0 (0)	10 (3.7)	1 (2.1)	1 (2.9)	0 (0)	1 (1.6)
I feel there is adequate technician staffing at my practice site to provide a safe environment for patient care	SA	4 (30.8)	25 (4.2)	129 (40.7)	8 (38.1)	44 (16.4)	11 (23.4)	6 (17.1)	5 (15.6)	16 (26.2)
	A	5 (38.5)	112 (19.0)	137 (43.2)	9 (42.9)	106 (39.4)	20 (42.6)	17 (48.6)	20 (62.5)	26 (42.6)
	N	2 (15.4)	96 (16.2)	24 (7.6)	1 (4.8)	29 (10.8)	6 (12.8)	4 (11.4)	2 (6.3)	9 (14.8)
	D	2 (15.4)	218 (36.9)	25 (7.9)	3 (14.3)	66 (24.5)	7 (14.9)	7 (20.0)	4 (12.5)	7 (11.5)
	SD	0 (0)	140 (23.7)	2 (0.6)	0 (0)	24 (8.9)	3 (6.4)	1 (2.9)	1 (3.1)	3 (4.9)
I feel there is adequate pharmacist staffing at my practice site to provide a safe environment for patient care	SA	5 (33.3)	34 (5.8)	139 (43.8)	9 (42.9)	57 (21.2)	10 (21.3)	7 (20.0)	3 (9.4)	15 (24.6)
	A	6 (40.0)	173 (29.3)	140 (44.2)	11 (52.4)	122 (45.4)	24 (51.1)	19 (54.3)	20 (62.5)	30 (49.2)
	N	2 (13.3)	117 (19.8)	19 (6.0)	0 (0)	33 (12.3)	6 (12.8)	2 (5.7)	4 (12.5)	8 (13.1)
	D	2 (13.3)	181 (30.6)	16 (5.0)	1 (4.8)	42 (15.6)	6 (12.8)	6 (17.1)	3 (9.4)	7 (11.5)
	SD	0 (0)	86 (14.6)	3 (0.9)	0 (0)	15 (5.6)	1 (2.1)	1 (2.9)	2 (6.3)	1 (1.6)
I feel the workload to staff ratio allows me to provide for patients in a safe manner	SA	4 (26.7)	19 (3.2)	91 (29.0)	8 (38.1)	45 (16.7)	7 (14.9)	4 (11.4)	3 (9.4)	13 (21.3)
	A	6 (40.0)	112 (19.0)	164 (52.2)	9 (42.9)	110 (40.9)	28 (59.6)	15 (45.7)	17 (53.1)	30 (49.2)
	N	4 (26.7)	109 (18.5)	31 (9.9)	3 (14.3)	52 (19.3)	4 (8.5)	7 (20.0)	7 (21.9)	10 (16.4)
	D	1 (6.7)	237 (40.2)	27 (8.6)	1 (4.8)	52 (19.3)	6 (12.8)	7 (20.0)	4 (12.5)	6 (9.8)
	SD	0 (0)	112 (19.0)	1 (0.3)	0 (0)	10 (3.7)	2 (4.3)	1 (2.9)	1 (3.1)	2 (3.3)
I feel pressured or intimidated to meet standards or metrics that may interfere with safe patient care at my practice site	SA	1 (6.7)	176 (29.7)	13 (4.1)	0 (0)	11 (4.1)	4 (8.5)	7 (20.6)	1 (3.1)	5 (8.2)
	A	3 (20.0)	207 (35.0)	41 (13.0)	4 (19.0)	52 (19.3)	8 (17.0)	6 (17.6)	5 (15.6)	10 (16.4)
	N	1 (6.7)	93 (15.7)	46 (14.6)	3 (14.3)	53 (19.7)	8 (17.0)	6 (17.6)	6 (18.8)	13 (21.3)
	D	5 (33.3)	81 (13.7)	118 (37.3)	5 (23.8)	97 (36.1)	18 (38.3)	8 (23.5)	15 (46.9)	21 (34.4)
	SD	5 (33.3)	35 (5.9)	98 (31.0)	9 (42.9)	56 (20.8)	9 (19.1)	7 (20.6)	5 (15.6)	12 (19.7)
I am given the opportunity to take lunch breaks or time away from the pharmacy in my practice	SA	7 (46.7)	44 (7.5)	88 (27.8)	11 (52.4)	66 (24.6)	23 (48.9)	15 (42.9)	8 (25.0)	22 (36.1)
	A	3 (20.0)	110 (18.6)	105 (33.1)	6 (28.6)	130 (48.5)	18 (38.3)	16 (45.7)	20 (62.5)	20 (32.8)
	N	2 (13.3)	56 (9.5)	36 (11.4)	1 (4.8)	29 (10.8)	4 (8.5)	3 (8.6)	2 (6.3)	11 (18.0)
	D	1 (6.7)	118 (20.0)	68 (21.5)	3 (14.3)	24 (9.0)	0 (0)	1 (2.9)	1 (3.1)	6 (9.8)
	SD	2 (13.3)	262 (44.4)	20 (6.3)	0 (0)	19 (7.1)	2 (4.3)	0 (0)	1 (3.1)	2 (3.3)
I am happy with my current practice site and working environment	SA	6 (40.0)	46 (7.8)	144 (45.6)	12 (57.1)	71 (26.6)	16 (34.0)	10 (28.6)	7 (21.9)	19 (31.1)
	A	6 (40.0)	146 (24.7)	120 (38.0)	4 (19.0)	126 (47.2)	22 (46.8)	14 (40.0)	18 (56.3)	29 (47.5)
	N	0 (0)	155 (26.3)	35 (11.1)	2 (9.5)	43 (16.1)	5 (10.6)	6 (17.1)	5 (15.6)	7 (11.5)
	D	3 (20.0)	160 (27.1)	14 (4.4)	3 (14.3)	25 (9.4)	3 (6.4)	3 (8.6)	1 (3.1)	4 (6.6)
	SD	0 (0)	83 (14.1)	3 (0.9)	0 (0)	2 (0.7)	1 (2.1)	2 (5.7)	1 (3.1)	2 (3.3)

a Given missing data, not all columns add to total N for that column.

b SA = Strongly Agree; A = Agree; N=Neutral D=Disagree; SD=Strongly Disagree.

c Amb Care = ambulatory care; Comm-Chain = chain community pharmacy; Comm-Indep = independent community pharmacy; Compound = compounding; LTC = long-term care.

When asked about adequate staffing to provide a safe environment for patient care, 23.2% of community chain pharmacists agreed or strongly agreed they have adequate pharmacy technician staffing; alternatively, about 55% of inpatient hospital pharmacists and 88% of independent community pharmacists agreed that they have adequate pharmacy technician staffing. The percentage of pharmacists who agreed/strongly agreed that pharmacist staffing is adequate to provide a safe environment for patient care ranged from 35.1% in the chain community pharmacy setting to 95.3% in the compounding setting. Similarly, when asked if the workload to staff ratio allows the pharmacist to provide for patients in a safe manner, between 22% (chain community) and 81% (compounding) of respondents agreed/strongly agreed.

About two-thirds (64.7%) of chain community pharmacists agreed or strongly agreed that they feel pressured or intimidated to meet standards or metrics that may interfere with safe patient care at their practice sites. The percentage of pharmacists who responded similarly in other settings ranged from 17.1% in independent community to 38.2% in mail order pharmacy settings. The percentage of pharmacists who agreed/strongly agreed they are given the opportunity to take lunch breaks or time away from the pharmacy in their practice ranged from 26.1% of chain community pharmacists to 88.6% of mail order pharmacists. Lastly, when asked to respond to the item, "I am happy with my current practice site and working environment," about one-third (32.5%) of chain community pharmacists agreed or strongly agreed, whereas at least two-thirds of pharmacists in every other setting indicated the same.

Analyses across pharmacist roles indicated that regional managers/directors/VPs and clinical/specialty pharmacists reported more positive perceptions across all workplace safety items as compared to staff pharmacists and relief pharmacists (p values <.05)(Table 3). About half of staff (47.4%), relief (51.3%), and managers/PICs (51.0%) agreed or strongly agreed they have time to complete their jobs in a safe and effective manner. Three-fourths of regional managers/directors/VPs and 72% of clinical/specialty pharmacists indicated the same. The extent to which respondents disagreed/strongly disagreed that staffing ratios allow them to provide for patients in a safe manner ranged from 10.7% for regional managers/directors/VPs to 38.6% for staff pharmacists. Overall, staff, relief, and PIC/manager pharmacists tended to respond similarly across all workplace safety items.

**Table 3:** Pharmacists' workplace perceptions by primary role (N=1391)^a^.

Item	Response^b^	Primary role^c^				
		Clinical/specialty pharmacist (*N* = 136)	PIC/manager (*N* = 443)	Regional Mgr/ director/VP (*N* = 28)	Relief pharmacist (*N* = 115)	Staff pharmacist (*N* = 669)
I feel I have adequate time to complete my job in a safe and effective manner	SA	29 (21.3)	64 (14.4)	9 (32.1)	16 (13.9)	75 (11.2)
	A	69 (50.7)	162 (36.6)	12 (42.9)	43 (37.4)	242 (36.2)
	N	16 (11.8)	60 (13.5)	4 (14.3)	18 (15.7)	88 (13.2)
	D	19 (14.0)	109 (24.6)	2 (7.1)	27 (23.5)	165 (24.7)
	SD	3 (2.2)	48 (10.8)	1 (3.6)	11 (9.6)	99 (14.8)
I feel that my employer has provided a work environment that allows for safe patient care	SA	35 (25.7)	112 (25.4)	13 (46.4)	22 (19.1)	119 (17.8)
	A	65 (47.8)	146 (33.1)	12 (42.9)	43 (37.4)	229 (34.2)
	N	18 (13.2)	63 (14.3)	2 (7.1)	19 (16.5)	103 (15.4)
	D	17 (12.5)	81 (18.4)	0 (0)	23 (20.0)	143 (21.4)
	SD	1 (0.7)	39 (8.8)	1 (3.6)	8 (7.0)	75 (11.2)
I feel there is adequate technician staffing at my practice site to provide a safe environment for patient care	SA	25 (18.7)	86 (19.5)	10 (35.7)	20 (17.4)	107(16.0)
	A	57 (42.5)	150 (33.9)	12 (42.9)	40 (34.8)	193 (28.8)
	N	20 (14.9)	54 (12.2)	2 (7.1)	17 (14.8)	82 (12.3)
	D	28 (20.9)	107 (24.2)	3 (10.7)	25 (21.7)	176 (26.3)
	SD	4 (3.0)	45 (10.2)	1 (3.6)	13 (11.3)	111 (16.6)
I feel there is adequate pharmacist staffing at my practice site to provide a safe environment for patient care	SA	27 (19.9)	103 (23.3)	13 (46.4)	22 (19.1)	114 (17.1)
	A	70 (51.5)	164 (37.0)	9 (32.1)	48 (41.7)	254 (38.0)
	N	16 (11.8)	69 (15.6)	3 (10.7)	14 (12.2)	91 (13.6)
	D	20 (14.7)	76 (17.2)	3 (10.7)	23 (20.0)	142 (21.3)
	SD	3 (2.2)	31 (7.0)	0 (0)	8 (7.0)	67 (10.0)
I feel the workload to staff ratio allows me to provide for patients in a safe manner	SA	24 (17.6)	69 (15.7)	8 (28.6)	15 (13.2)	78 (11.7)
	A	63 (46.3)	153 (34.8)	15 (53.6)	41 (36.0)	220 (33.0)
	N	23 (16.9)	73 (16.6)	2 (7.1)	19 (16.7)	111 (16.6)
	D	24 (17.6)	108 (24.5)	3 (10.7)	32 (28.1)	175 (26.2)
	SD	2 (1.5)	37 (8.4)	0 (0)	7 (6.1)	83 (12.4)
I feel pressured or intimidated to meet standards or metrics that may interfere with safe patient care at my practice site	SA	5 (3.7)	74 (16.7)	0 (0)	15 (13.0)	124 (18.6)
	A	29 (21.3)	111 (25.1)	5 (17.9)	21 (18.3)	171 (25.6)
	N	23 (16.9)	73 (16.5)	4 (14.3)	25 (21.7)	105 (15.7)
	D	51 (37.5)	108 (24.4)	8 (28.6)	37 (32.2)	164 (24.6)
	SD	28 (20.6)	76 (17.2)	11 (39.3)	17 (14.8)	104 (15.6)
I am given the opportunity to take lunch breaks or time away from the pharmacy in my practice	SA	41 (30.4)	76 (17.2)	15 (53.6)	19 (16.5)	133 (19.9)
	A	63 (46.7)	116 (26.2)	8 (28.6)	26 (22.6)	215 (32.2)
	N	10 (7.4)	54 (12.2)	4 (14.3)	16 (13.9)	62 (9.3)
	D	13 (9.6)	72 (16.3)	1 (3.6)	32 (27.8)	104 (15.6)
	SD	8 (5.9)	124 (28.1)	0 (0)	22 (19.1)	154 (23.1)
I am happy with my current practice site and working environment	SA	44 (32.4)	114 (25.8)	15 (53.6)	24 (20.9)	134 (20.2)
	A	64 (47.1)	146 (33.0)	11 (39.3)	43 (37.4)	221 (33.2)
	N	16 (11.8)	82 (18.6)	1 (3.6)	31 (27.0)	130 (19.5)
	D	10 (7.4)	74 (16.7)	1 (3.6)	11 (9.6)	120 (18.0)
	SD	2 (1.5)	26 (5.9)	0 (0)	6 (5.2)	60 (9.0)

a Given missing data, not all columns add to total N for that column.

b SA = Strongly Agree; A = Agree; N=Neutral D=Disagree; SD=Strongly Disagree.

c PIC = pharmacist in charge; Mgr = manager; VP = vice president.

Average hours per shift was inversely correlated with favorable workplace safety perceptions (Table 4). Average hours per week was also inversely correlated with all workplace perceptions except for the extent to which pharmacists are given the opportunity to take lunch breaks or time away from the pharmacy.

**Table 4:** Correlation between hours worked and workplace perceptions (N=1391)^a^.

	Hours per shift	Hours per week
I feel I have adequate time to complete my job in a safe and effective manner	−0.200⁎⁎	−0.090⁎
I feel that my employer has provided a work environment that allows for safe patient care	−0.152⁎⁎	−0.078⁎
I feel there is adequate technician staffing at my practice site to provide a safe environment for patient care	−0.183⁎⁎	−0.094⁎⁎
I feel there is adequate pharmacist staffing at my practice site to provide a safe environment for patient care	−0.181⁎⁎	−0.102⁎⁎
I feel the workload to staff ratio allows me to provide for patients in a safe manner	−0.189⁎⁎	−0.057⁎
I feel pressured or intimidated to meet standards or metrics that may interfere with safe patient care at my practice site	0.120⁎⁎	0.089⁎
I am given the opportunity to take lunch breaks or time away from the pharmacy in my practice	−0.211⁎⁎	−0.031
I am happy with my current practice site and working environment	−0.174⁎⁎	−0.062⁎

⁎ p < .05.

⁎⁎ p < .001.

a Spearman's rho correlation.

## 4 Discussion

From a societal perspective, providing safe health care – regardless of the environment – is the expectation. Ideally, 100% of pharmacists would perceive their workplace to provide an environment that prioritizes and allows for safe patient care. While we found no settings in which 100% of pharmacists had positive perceptions of their workplace related to patient safety, a large majority of respondents in ambulatory care, independent community, compounding, and outpatient hospital pharmacy settings reported positive perceptions of the workplace and patient safety. Relatively fewer pharmacists practicing in an inpatient hospital, long-term care, and mail order settings reported positive patient safety workplace perceptions, but overall, perceptions were still favorable in these settings. However, pharmacists in chain community pharmacies were outliers compared to their peers in other practice settings.

Overall, our results either affirm findings in peer-reviewed literature and lay press investigations in the US or indicate that perceptions are getting more negative in some practice settings. The 2019 National Pharmacist Workforce Study found that about 50% of chain community pharmacists fear a patient will be harmed due to a medication error.^24^ Additionally, over 90% of chain community pharmacists reported high or excessively high workloads, the highest percent of any practice setting studied. The National Pharmacist Workforce Study authors stated, "There were significant drops in the amount of control pharmacists felt in the community pharmacy environments [as compared to 2014 data]".^24^ All of these findings from the 2019 national study align with our findings. Using survey items from the Oregon Board of Pharmacy study, Tsao et al.^21^ in 2013 found that 28% of British Columbia pharmacists surveyed disagreed or strongly disagreed that their employer provides a work environment that is conducive to safe and effective primary care. Whereas no more than 20% of respondents in non-chain settings in our study disagreed or strongly disagreed that their employer has provided a work environment that allows for safe patient care, 50.2% of chain community pharmacists responded negatively to the item.

While the aforementioned *New York Times* article brought renewed attention to the chain community work environment, this is not the first time major press articles have done so.^18^ The *USA Today*, for example, published a 3-day series on pharmacy work environments and medical errors 12 years ago, with an emphasis on chain community pharmacy settings.^25^ In the series, pharmacy workload, volume, staffing, and errors were reported as problematic in chain pharmacy settings. The Institute for Safe Medication Practices (ISMP) responded to the *USA Today* series and made several points about the community pharmacy work environment that are just as, if not more, relevant today.^26^ In their summary, ISMP called for increased communication (i.e., counseling) with patients, improved communication with other health care providers, manageable, safe workloads in pharmacies, reduction of prescription volume-based incentives, and increases in safety-based incentives, in addition to other statements.

Our study focused specifically on perceptions of the workplace and patient safety. It is unclear if pharmacists' perceptions reflect reality (i.e., the perceived unsafe workplace is indeed unsafe as evidenced by higher error rates, lesser quality patient care, and vice versa). What if a pharmacy or corporation expresses a commitment to an organizational culture of safety but the pharmacists practicing within the pharmacy perceive a workplace that does not allow for safe patient care? To that point, in our study, more favorable perceptions of the work environment were noted by pharmacists in administrative positions as compared to those in staffing and PIC roles. Of particular importance is the extent to which the work environment (as opposed to the actions or inactions of a pharmacist regardless of setting characteristics) actually contributes to or causes errors and downstream patient harm. Establishing causality is difficult within one company and near impossible across multiple private corporations. Research findings specific to the causal nature of the busyness of pharmacies (prescriptions filled/dispensed per hour) and error rates are both limited and mixed.^13^ The causal nature of pharmacists' perceptions and patient safety is limited more so. Regardless, in a work environment where safety is not perceived to be valued or prioritized, whether it is or not, the moral distress experienced by pharmacists as well as a lack of psychological safety to advocate for safe patient care in the workplace could contribute to negative downstream pharmacist and patient consequences. Based on our results, there is a need for chain pharmacies, and arguably other settings as well, to communicate in the language of patient safety. A human factors and systems engineering approach - the approach proposed by the US Institute of Medicine - could facilitate transparency and communication across levels of the system and roles.^27^^,^^28^ A model such as the SEIPS model could facilitate communication and solutions that improve patient safety and employee and organizational outcomes.^29^

While mechanisms exist to report medication-related errors (e.g., FDA's MedWatch, ISMP's Medication Errors Reporting Program), and many states have regulations or statutes requiring errors and patient harm be reported, the ability to distinguish between errors/incidents resulting directly from the workplace environment versus those that would have happened regardless of the workplace environment is difficult. James et al.^13^ mentioned that studies examining dispensing errors focus on unprevented errors much more frequently than prevented errors. The relationship between pharmacists' workplace patient safety perceptions and pharmacists' perceptions of their ability to prevent or catch errors warrants investigation.

In the investigation conducted by the *Chicago Tribune*, independent pharmacies failed to counsel patients and contact prescribers about a drug interaction more so than their chain pharmacist colleagues.^17^ These findings could be interpreted as provision of unsafe patient care and resulted in patient safety efforts across independent and chain pharmacies alike. On the other hand, our study found very little patient safety concern among pharmacists practicing in independent pharmacy settings, especially compared to those practicing in chain settings. It could be that larger pharmacy corporations have the science of safety informed systems-level infrastructure (e.g., standardization of roles across pharmacy technicians and pharmacists) that independent pharmacies do not protect patients whether chain pharmacists realize it. Such approaches are evidence-based, science of safety approaches.^30^ Or, perhaps the stress and workload of chain pharmacists are simply assumed to result in downstream patient harm when in fact, it does not, or at least does not to the extent pharmacists in the environment may perceive it does. In our study, for example, regional managers, directors, and vice presidents reported more favorable perceptions of the work environment as compared to pharmacists in the trenches of practice. Perhaps their perceptions are more accurate. The dissonance between individual CVS pharmacists' perceptions of the workplace and CVS' corporate response in the *New York Times* investigation that errors are regrettable but rare reflects this assertion.^18^

An approach that could foster patient safety is establishing a mechanism to include one or more patient safety metrics in pharmacy ratings and key performance indicators and link patient safety with reimbursement. This aligns with previously mentioned ISMP recommendations.^26^ For example, community pharmacies are evaluated by the Centers for Medicare and Medicaid Services (CMS) via health plans using star ratings. To date, patient safety metrics for community pharmacies have been framed around evidence-based, disease-specific medication use (e.g., statin use in patients with diabetes). Such measures fail to capture errors at the point of dispensing or the safety of the work environment. Such a measure or indicator would undoubtedly be difficult to validate and would necessitate that pharmacist errors (unprevented, prevented, or both) are reported in a manner that is comprehensive, non-punitive, and transparent across companies big and small. A patient-centered outcomes approach to this task – including engaging patients in the process – is warranted.

While more data are warranted to understand the relationship between the pharmacist work environment and patient safety, decisions are often made in the absence of sufficient data. Legislators have proposed statutes that place limits on the number of prescriptions that can be verified by a pharmacist per shift.^31^^,^^32^ Boards have established limits on the number of technicians a pharmacist can supervise at one time,^33^ and the National Association of Boards of Pharmacy has resolved to "assist the state boards of pharmacy to regulate, restrict, or prohibit the use in pharmacies of performance metrics or quotas that are proven to cause distractions and unsafe environments for pharmacists".^34^ The extent to which policies are enforceable and the extent to which they result in increased patient safety is unknown. The current pharmacist labor market complicates matters as pharmacists may be less inclined to report violations and abide by statutes or regulations within their practice sites, fearing they can easily be replaced. Specific, enforceable retaliation-proof pharmacist protection via whistleblower statutes could perhaps give pharmacists a voice by fostering reporting of unsafe practice environments.

### 4.1 Limitations

Our study had several limitations that warrant discussion. First, despite adapting the survey instrument from previous literature, the lack of a rigorous evaluation of survey instrument reliability and validity is a limitation. Results should be interpreted with this limitation in mind. Second, it is not possible to determine the extent to which the results obtained in this study reflect general perceptions of all actively licensed pharmacists in the state or beyond its borders. The sample represents about 10% of all licensed pharmacists in the state. However, only a subset of actively licensed pharmacists actually practices in the state. It is therefore unclear what percentage of pharmacists practicing in Tennessee are represented by this study. Third, social desirability could have influenced pharmacists' responses, perhaps differently depending on the setting in which the survey was completed. For example, pharmacists completing the survey in a large audience might have responded differently than a pharmacist completing the survey in the presence of a Board investigator during a practice site visit. Finally, it is possible that pharmacists could have completed the survey more than once. Regardless of where the survey was completed, Board representatives asked pharmacists only to complete the survey if they had not previously done so.

## 5 Conclusion

Chain community pharmacists in Tennessee reported less positive perceptions of the work environment and patient safety as compared to pharmacists in all other practice settings studied. The extent to which policy should address workplace safety and how it can do so successfully if desired is complex. State boards of pharmacy are wrestling with how best to proactively protect public health by regulating pharmacists and pharmacy practice sites, knowing that failure to do so will likely result in legislative intervention. Additional research is warranted to understand better the relationship between pharmacist perceptions and quantifiable patient safety metrics (e.g., dispensing errors).

## Disclaimer

The content is solely the responsibility of the authors. The conclusions derived from the data are not the opinions of the Tennessee Board of Pharmacy.

## Declaration of Competing Interest

The authors declare that they have no known competing financial interests or personal relationships that could have appeared to influence the work reported in this paper.

## Appendix A Supplementary data

Survey Instrument Image 1
