# Supplementary material for: An analysis of pharmacists' workplace patient safety perceptions across practice setting and role characteristics
Source: Explor Res Clin Soc Pharm. 2021 Jun 29;2:100042. doi: 10.1016/j.rcsop.2021.100042 (PMC9031369; doi:10.1016/j.rcsop.2021.100042)
Supplement: Supplementary file 1 — Survey Instrument [file mmc1.docx]

1. I feel I have adequate time to complete my job in a safe and effective manner.

Strongly Agree Agree Neutral Disagree Strongly Disagree

1. I feel that my employer has provided a work environment that allows for safe patient care.

Strongly Agree Agree Neutral Disagree Strongly Disagree

1. I feel there is adequate technician staffing at my practice site to provide a safe environment for patient care.

Strongly Agree Agree Neutral Disagree Strongly Disagree

1. I feel there is adequate pharmacist staffing at my practice site to provide a safe environment for patient care.

Strongly Agree Agree Neutral Disagree Strongly Disagree

1. I feel the work load to staff ratio allows me to provide for patients in a safe manner.

Strongly Agree Agree Neutral Disagree Strongly Disagree

1. I feel pressured or intimidated to meet standards or metrics that may interfere with safe patient care at my practice site.

Strongly Agree Agree Neutral Disagree Strongly Disagree

1. I am given the opportunity to take lunch breaks or time away from the pharmacy in my practice

Strongly Agree Agree Neutral Disagree Strongly Disagree

1. I am happy with my current practice site and working environment.

Strongly Agree Agree Neutral Disagree Strongly Disagree

1. What is your primary role as a pharmacist?

Staff pharmacist Clinical/specialty PIC/manager

Relief pharmacist Regional manager/director/VP

1. What is your primary practice site?

In-patient hospital Out-patient hospital Community (independent)

Community (chain) Long term care Ambulatory care

Compounding Mail order Other

1. On average how many hours do you work per shift?

<5.9 hrs 6-7.9 hrs 8-9.9 hrs 10-12.9 hrs >13 hrs

1. On average how many hours do you work per week?

<30 hrs 30-39.9 hrs 40-49.9 hrs 50-59.9 hrs >60 hrs

1. Any additional comments that you think would be helpful to the Board:
